# Next Place Prediction Based on Spatiotemporal Pattern Mining of Mobile Device Logs

**DOI:** 10.3390/s16020145

**Published:** 2016-01-23

**Authors:** Sungjun Lee, Junseok Lim, Jonghun Park, Kwanho Kim

**Affiliations:** 1 Department of Industrial Engineering, Seoul National University, 1 Gwanak-ro, Gwanak-gu, Seoul 08826, Korea; zaregn81@snu.ac.kr (S.L.); kasia05@snu.ac.kr (J.L.); 2 Department of Industrial & Management Engineering, Incheon National University, 119 Academy-ro, Yeonsu-gu, Incheon 22012, Korea; khokim@inu.ac.kr

**Keywords:** next place prediction, movement patterns, spatiotemporal patterns, Markov chain, gapped sequence mining

## Abstract

Due to the recent explosive growth of location-aware services based on mobile devices, predicting the next places of a user is of increasing importance to enable proactive information services. In this paper, we introduce a data-driven framework that aims to predict the user’s next places using his/her past visiting patterns analyzed from mobile device logs. Specifically, the notion of the spatiotemporal-periodic (STP) pattern is proposed to capture the visits with spatiotemporal periodicity by focusing on a detail level of location for each individual. Subsequently, we present algorithms that extract the STP patterns from a user’s past visiting behaviors and predict the next places based on the patterns. The experiment results obtained by using a real-world dataset show that the proposed methods are more effective in predicting the user’s next places than the previous approaches considered in most cases.

## 1. Introduction

Owing to the recent exponential growth of location-aware services based on mobile devices, such as smart phones, smart watches and tablet PCs, predicting a user’s next place becomes an important research topic in both academia and industry [1,2,3,4,5]. This problem concentrates on predicting a place that will be visited by a user in advance before she/he arrives, on the basis of the user’s past visiting behaviors inferred through utilizing sensors, such as Global Positioning System (GPS) and wireless fidelity(WiFi) sensor, that are commonly available in modern mobile devices.

When the level of geographical granularity for prediction comes into consideration, a more precise level is desired to enable further sophisticated services. Through discovering the next places at the level of users’ daily lives, such as local shops and school cafeterias, various customized applications can be enabled, including recommendation of tailored information, such as automated reservation and personalized advertisements [6,7,8].

To predict a user’s next place, three types of patterns, namely sequential, temporal sequential and periodic patterns, have been intensively studied. Mobile sequential patterns have been utilized to predict next places based on frequently-observed sequential patterns of the places visited [9]. Gambs *et al.* proposed a modified version of a Markov chain to predict next places by analyzing mobile movement behaviors [10]. Similarly, Alavarez-Garcia *et al.* and Jeung *et al.* employed a hidden Markov chain-based method to infer a user’s final place [11,12].

Gidófalvi *et al.* extended the Markov chain-based approach for predicting next places in a continuous manner by adopting an inhomogeneous continuous-time Markov model [13,14]. Rodriguez-Carrion *et al.* suggested a light version of the Lempel-Ziv (LZ) based prediction algorithm to perform predictions on mobile devices [15,16]. Morzy and Pei *et al.* proposed a rule-based approach that discovers associations between an individual user and a place through utilizing a modified *a priori* algorithm [17,18,19].

Furthermore, several attempts have been made to enhance the performances of next place prediction by considering both spatial and temporal aspects. Giannotti *et al.* classified users’ moving intentions into geographically-triggered and temporally-triggered intentions in terms of place and time [20]. Lu *et al.* proposed a methodology for mining two types of trajectory patterns, periodic behavior and swarm pattern [21]. By using a nonlinear time series analysis, Scellato *et al.* attempted to additionally consider arrival time in predicting next places [22]. Wang and Prabhala proposed a user-specific periodicity model based on each user’s visiting history [23].

While the previous work mainly focused on the problem of predicting the user’s next locations in terms of cell IDs [15,16,24] or at the levels of intra-city or inter-cities [9,10,14,22,25,26], the geographical granularity considered in this paper is at the level of people’s daily lives (e.g., buildings). When applied to such a fine level of location granularity, the previous approaches suffer from one or more limitations due to the following unique characteristics of the next place prediction problem discussed in this paper.

First, mobile device logs are likely to contain much noise and missing data related to users’ past visits, which is caused by various reasons, such as measurement errors, wireless connection problems or unpowered mobile devices. Dealing with such noise and missing data is crucial, since they make it difficult to achieve accurate parameter estimation and rule generation, leading to unrealistic predictions of next places eventually. Moreover, as the geographical granularity becomes finer, the impact of such error-prone data on the performance of a prediction method becomes more severe.

Next, compared to the cases with coarse location granularity, there is a larger amount of irregular visits in the past history of a user in the considered problem, which makes it even more difficult for a prediction model to accurately identify the user’s visiting patterns. For instance, various irregular visits, such as going shopping or to movies, are frequently found between the regular visits of going to and returning from work. As a result, if those irregular visits are simply ignored, prediction models often fail to capture the patterns hidden among them.

Finally, it is necessary to be able to predict the next place for a user by utilizing only a small amount of observations available for the user, since collecting a user’s mobile device log is usually a time-consuming and costly task. Accordingly, the methods, such as rule mining and decision tree, that require a significant amount of history information for prediction do not appear to be a viable option when time and cost are an issue.

Motivated by the above remarks, we attempt to develop a novel framework that aims to predict a user’s next place based on the user’s past visiting behaviors through considering periodicity in addition to time and location. To address the three challenges mentioned above, the proposed framework maps the individual visit of a user to one of the visiting patterns by utilizing the pattern extraction algorithms and the pattern similarity function proposed in this research.

The proposed framework constructs spatiotemporal (ST) trajectories, each of which represents a sequence of stays in terms of place and time, from a limited amount of past visit data for each user. Spatiotemporal-periodic (STP) patterns are then extracted from the user’s ST trajectories by the proposed STP extraction algorithm. The algorithm searches for STP patterns through considering both occurrence frequencies and associations with ST trajectories with respect to time for effective recognition of irregular or new visits as STP patterns. In particular, we employ a smoothing function to deal with the noisy and missing data.

Subsequently, STP trajectories are built by mapping each ST trajectory to an STP pattern that is most similar to the trajectory among the extracted STP patterns. Basing on gapped sequence mining [27], the proposed framework is able to identify user’s sporadic visits in her/his daily life through constructing gapped STP (GSTP) trajectories that allow gaps to accommodate irregular visits that cannot be specified in advance. The next place visited by a user is then predicted by the proposed prediction algorithm based on the user’s current and recent visits.

This paper is organized as follows: In Section 2, the details of the proposed methods are described. In Section 3, the data collection details and experimentation results are described. The conclusions are presented in Section 4.

## 2. Proposed Framework

**Figure 1:**
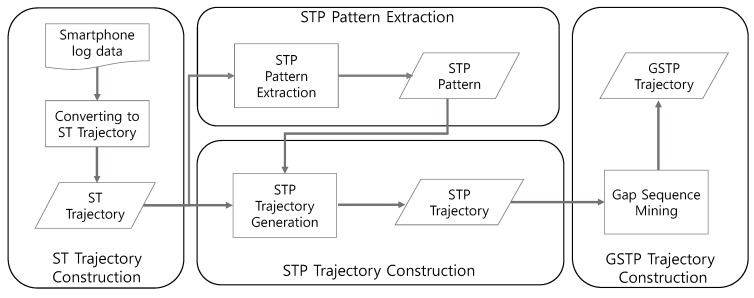
The training process of the proposed approach for computing gapped spatiotemporal-periodic (GSTP) trajectories.

In this section, we describe in detail how the proposed framework extracts GSTP trajectories and predicts the user’s next places through considering sequential, temporal and periodic characteristics of a mobile device log. Figure 1 illustrates the overall training process of the proposed framework that consists of four steps to generate GSTP trajectories. The training process proceeds as follows.

First, an ST trajectory, defined as a sequence of stays in which each stay is represented in terms of a place visited, and the arrival and departure time, as well as the day of week for the visit, is constructed from raw data. Second, we extract STP patterns from ST trajectories to capture periodic revisits by taking periodicity into consideration. The existence of an STP pattern for a user indicates that the user tends to periodically revisit a particular place at a specific time associated with the pattern.

Next, in the STP trajectory construction step of Figure 1, ST trajectories are mapped into a sequence of the extracted STP patterns, named the STP trajectory, based on the similarity between an STP pattern and an element of an ST trajectory. Finally, gap-constrained sequential pattern mining is applied to the STP trajectories to construct a user’s GSTP trajectory that allows unobserved places in the user’s STP trajectories. The generated GSTP trajectories from the training process are then used for prediction of the next place when the user’s most recent STP trajectory data are provided as test data. The detailed descriptions are presented in the following sections.

### 2.1. WiFi-Based Place Identification

We employ a WiFi fingerprint-based localization method [28] for extracting the places visited from a user’s mobile device log. It is well known that this method has advantages over GPS-based approaches when tracking and identifying people’s movements in indoor environments, particularly in urban areas, and the method also provides several benefits in terms of energy efficiency, compared to the GPS-based ones, as it utilizes WiFi sensor data.

The localization method requires a WiFi fingerprinting database, containing WiFi access point (AP) data, each of which consists of a place, *p*, the basic service set identifier (BSSID) and a range of received signal strengths (RSSI) observed at *p*. The database is used to infer a user’s visit to a place by matching WiFi APs with those in the database according to BSSIDs and their RSSI ranges.

Table 1 shows an example for the WiFi fingerprint-based localization method. The example shows a WiFi fingerprinting database, raw WiFi data and the localization result in Table 1a–c, respectively, where distinct places are indexed. As an example, for an instance observed at 15:00 on 11 November 2013 in Table 1b, the place is identified as $p_{2}$, since only $p_{2}$ in the database has the matching BSSIDs and RSSI values in range with those of the instance.

**Table 1:** Examples of: (**a**) the WiFi fingerprinting database; (**b**) raw WiFi data; and (**c**) places identified by applying a fingerprinting-based localization method. BSSID, basic service set identifier.

(a)					
**Place**	**BSSID**	**RSSI**	**Place**	**BSSID**	**RSSI**
$p_{1}$	BSSID_4	−48 to −40	$p_{2}$	BSSID_3	−33 to −23
$p_{1}$	BSSID_5	−39 to −31	$p_{3}$	BSSID_1	−28 to −18
$p_{2}$	BSSID_1	−57 to −49	$p_{3}$	BSSID_4	−72 to −61
$p_{2}$	BSSID_2	−40 to −29	$p_{3}$	BSSID_6	−63 to −53
(**b**)					
**Timestamp**		**BSSID**		**RSSI**	
11 November 2013 15:00		BSSID_1		−55	
		BSSID_2		−34	
		BSSID_3		−22	
11 November 2013 15:45		BSSID_4		−44	
		BSSID_5		−33	
11 November 2013 15:55		BSSID_1		−49	
		BSSID_2		−38	
		BSSID_3		−26	
...					
(**c**)					
**Timestamp**			**Place**		
11 November 2013 15:00			$p_{2}$		
11 November 2013 15:45			$p_{1}$		
11 November 2013 15:55			$p_{2}$		
11 November 2013 16:25			$p_{2}$		
11 November 2013 17:15			$p_{3}$		
11 November 2013 19:00			$p_{3}$		
11 November 2013 19:15			$p_{2}$		
11 November 2013 20:00			$p_{2}$		
...					

More specifically, the observation of BSSID_1 at 15:00 on 11 November 2013 in Table 1b indicates that the place can be either $p_{2}$ or $p_{3}$ according to Table 1a, but only the RSSI range of $p_{2}$ contains -55, the observed RSSI value. Similarly, the observations of BSSID_2 and BSSID_3 at 15:00 on 11 November 2013 also indicates that place must be $p_{2}$, and therefore, the place at 15:00 on 11 November 2013 is inferred to be $p_{2}$. Through repeating this process for all of the instances in raw WiFi data collected, the places visited by a user, as well as the corresponding timestamps can be constructed. An example of the localization result is shown in Table 1c, where each timestamp is interpreted as the time when the user arrived at a place, indicating that the user was at $p_{2}$ from 15:00 to 15:45 on 11 November 2013, for instance.

### 2.2. ST Trajectory Construction

Once the places and their associated timestamps are identified, the ST trajectory of the *i*-th day, $T_{i}$, is constructed. $T_{i}$ denotes a sequence of stays, and each stay, $T_{i,j}$, is defined as a four-tuple, $\left(p,t_{s},t_{f},d\right)$, where $t_{s}$ and $t_{f}$, respectively, represent the start and finish time of the stay in minutes, and d∈{Mo,Tu,We,Th,Fr,Sa,Su} is the day of the week for $T_{i}$. $T_{i,j}$’s are ordered chronologically in $T_{i}$.

$T_{i,j}$ is identified from the log containing the localization result by grouping consecutive logs corresponding to the same place while preserving the ascending order of the timestamps. $t_{s}$ and $t_{f}$ are determined to be the start and the finish time of the group, respectively, and *p* is set to the place of the group. Subsequently, $T_{i}$ is constructed from identified $T_{i,j}$’s according to the value of *i* of $T_{i,j}$.

Table 2 shows an example of ST trajectories constructed from the data in Table 1c, assuming that 11 November 2013 is Monday. The first stay, $T_{1,1}$, shown in Table 2, corresponds to the first instance of Table 1c, since the places of the first and second instance of Table 1c are different. As a result, $t_{s}$ and $t_{f}$ for $T_{1,1}$ are set to the timestamps of the first and second instance of Table 1c, respectively, leading to $T_{1,1}=\left(900,945,p_{2},Mo\right)$. Since the minutes are measured from the beginning of a day, the user is inferred to have stayed at $p_{2}$ between 15:00 to 15:45 on Monday. The other stays are generated similarly from Table 1c, and $T_{i}$’s are constructed as shown in Table 2.

**Table 2:** ST trajectory examples.

$T_{i}$	$T_{i,j}$	$\left(t_{s},t_{f},p,d\right)$	$T_{i}$	$T_{i,j}$	$\left(t_{s},t_{f},p,d\right)$
$T_{1}$	$T_{1,1}$	$\left(900,945,p_{2},Mo\right)$	$T_{15}$	$T_{15,1}$	$\left(895,1080,p_{2},Mo\right)$
	$T_{1,2}$	$\left(945,955,p_{1},Mo\right)$		$T_{15,2}$	$\left(1110,1125,p_{1},Mo\right)$
	$T_{1,3}$	$\left(955,1035,p_{2},Mo\right)$		$T_{15,3}$	$\left(1165,1230,p_{2},Mo\right)$
	$T_{1,4}$	$\left(1035,1155,p_{3},Mo\right)$		$T_{15,4}$	$\left(1240,1320,p_{4},Mo\right)$
	$T_{1,5}$	$\left(1155,1260,p_{2},Mo\right)$	...		
...					
$T_{8}$	$T_{8,1}$	$\left(900,1140,p_{4},Mo\right)$			
	$T_{8,2}$	$\left(1140,1280,p_{3},Mo\right)$			
...					

### 2.3. STP Pattern Extraction

In this paper, we consider a weekly periodicity for extracting STP patterns of a user, as most people have weekly visiting patterns [29]. Extraction of the STP patterns from ST trajectories consists of three steps: grouping ST trajectories based on weekly periodicities, computing the probabilities of a stay according to its periodicity group membership and generating STP patterns from the probabilities.

Specifically, ST trajectories are grouped according to the day of the week contained in $T_{i}$ to accommodate the weekly periodicity of user’s movements. For each group of ST trajectories, the probability of a stay is computed through examining whether or not a user has visited a place at a specific time based on the arrival and departure time. Then, STP patterns are extracted by finding time segments that exceed a certain threshold in terms of the probability. Detailed description of each step is given in the following.

#### 2.3.1. ST Trajectory Grouping for Periodicity Identification

In order to take the periodicity into account, ST trajectories are grouped based on a weekly periodicity, denoted as D∈D, where D denotes a set of all possible combinations of the days of the week, {Mo,Tu,We,Th,Fr,Sa,Su}. For instance, *D* = {Mo,We} represents a periodicity of visits that tend to be made on every Monday and Wednesday. We let $T_{D}$ be the set of $T_{i}$’s containing $T_{i,j}$ whose *d* belongs to *D*. For instance, if *D* = {Mo}, $T_{\left\{Mo\right\}}$ formed from Table 2 can be expressed as $\left\{T_{i}|i=7\left(x-1\right)+1,x\in N\right\}$, where N is the set of positive integers.

#### 2.3.2. Computing the Probability of a Stay

Given *D*, $T_{D}$ is used for computing the probability of a stay at place *p* at a discrete time, $t\in \{1,...,t_{max}\}$, denoted as $q_{D,p,t}$, where *t* and $t_{max}$, respectively, are the time since the beginning of a day and the time at the end of the day. Both *t* and $t_{max}$ are measured in minutes, ranging from 1 to 1440, and accordingly $t_{max}$ = 1440.

A procedure for calculating $q_{D,p,t}$ based on counting the number of stays in the ST trajectories that belong to $T_{D}$ is shown in Algorithm 1 in which $P(T_{D})$ is a function that returns the set of places included in the ST trajectories contained in $T_{D}$. In Line 2, the temporary variable, $Q_{D,p,t}\left(T_{i}\right)$, that stores the probability of a stay for $T_{i}$ is initialized to zero for all *t*. In Lines 5 to 11, $Q_{D,p,t}\left(T_{i}\right)$ becomes one if there exists a stay $T_{i,j}=\left(p,t_{s},t_{f},d\right)\in T_{i}$, such that $t_{s}\leq t\leq t_{f}$, and set to the maximum between its current value and the result of smoothing, otherwise. Finally, $q_{D,p,t}$ is computed by averaging $Q_{D,p,t}\left(T_{i}\right)$ across $T_{i}$’s in $T_{D}$, in Line 15.

Linear smoothing is applied to $q_{D,p,t}$ to accommodate the variability of a stay, as well as noise in the raw WiFi data. $\lambda_{s}$ is a slope parameter that determines the penalty amount for the stays that are not exactly matched in terms of time. The penalty is proportional to the distance between *t* and $t_{s}$ or between *t* and $t_{f}$, increasing $Q_{D,p,t}\left(T_{i}\right)$ as *t* goes near $t_{s}$ or $t_{f}$, but only up to $Q_{D,p,t_{s}}\left(T_{i}\right)$ or $Q_{D,p,t_{f}}\left(T_{i}\right)$, respectively.
**Algorithm 1:** Algorithm for Calculating the Probability of a Stay.
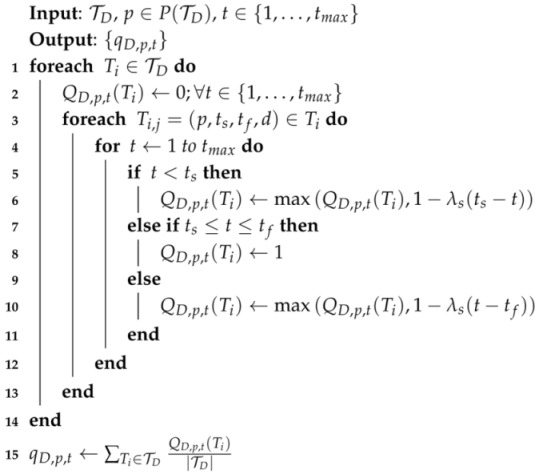


**Figure 2:**
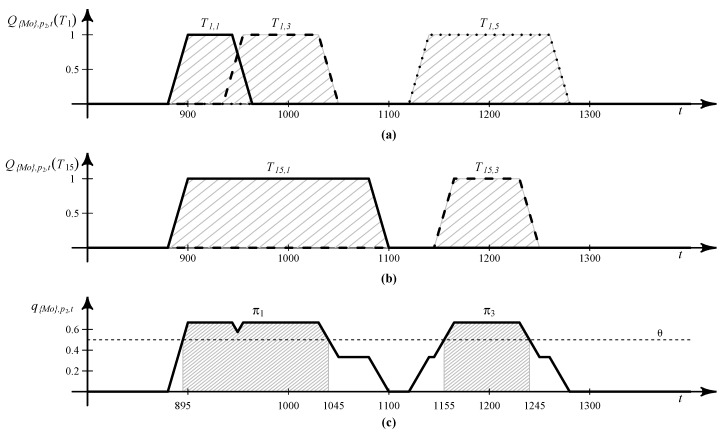
Examples illustrating the calculation of: (**a**) $Q_{\left\{Mo\right\},p_{2},t}\left(T_{1}\right)$; (**b**) $Q_{\left\{Mo\right\},p_{2},t}\left(T_{15}\right)$; and (**c**) $q_{\left\{Mo\right\},p_{2},t}$.

Figure 2a,b respectively shows the calculation results of $Q_{\left\{Mo\right\},p_{2},t}\left(T_{1}\right)$ and $Q_{\left\{Mo\right\},p_{2},t}\left(T_{15}\right)$ over time, based on Table 2 when D={Mo}, $T_{D}=\left\{T_{1},T_{8},T_{15}\right\}$, and $\lambda_{s}=0.05$. Note that $Q_{\left\{Mo\right\},p_{2},t}\left(T_{8}\right)\;=\;0$ for all *t*, since $T_{8}$ does not include any stay at $p_{2}$, although $T_{8}\in T_{D}$. Figure 2c shows the plot for $q_{\left\{Mo\right\},p_{2},t}$ based on $T_{1,1}$, $T_{1,3}$, $T_{1,5}$, $T_{15,1}$ and $T_{15,3}$ for each *t*, where trapezoid shapes are attributed to the application of smoothing to the stay probabilities.

Specifically, the value of $q_{\left\{Mo\right\},p_{2},t}$ is computed as follows: First, we consider $T_{1,1}$ and compute $Q_{\left\{Mo\right\},p_{2},t}\left(T_{1}\right)$. Since $t_{s}$ and $t_{e}$ for $T_{1,1}$ are 900 and 945, respectively, $Q_{\left\{Mo\right\},p_{2},t}\left(T_{1}\right)=1$ when t∈[900,945]. For other *t*’s, the linear smoothing function in Algorithm 1 is applied. As a result, $Q_{\left\{Mo\right\},p_{2},t}\left(T_{1}\right)=0$ when t<880 or t>965, $Q_{\left\{Mo\right\},p_{2},t}\left(T_{1}\right)=1-0.05\left(900-t\right)$ when t∈[880,900) and $Q_{\left\{Mo\right\},p_{2},t}\left(T_{1}\right)=1-0.05\left(t-945\right)$ when t∈(945,965]. The probabilities of stays at $p_{2}$ of $T_{1,3}$ and $T_{1,5}$ are computed similarly, and the results are shown in Figure 2a. Subsequently, we repeat the above probability calculations for all $T_{i}\in T_{D}$ and set $q_{\left\{Mo\right\},p_{2},t}$ as the average of probabilities computed for $T_{1}$ and $T_{15}$, resulting in the thick line in Figure 2c.

#### 2.3.3. Extracting STP Patterns

After computing $q_{D,p,t}$ for all D,p and *t*, we proceed to compute the set of STP patterns for *D*, denoted as $\Pi_{D}$. We define each STP pattern $\pi \in \Pi_{D}$ as a triplet, $\left(p,\tau_{s},\tau_{f}\right)$, where $\tau_{s}$ and $\tau_{f}$, respectively, stand for the start and finish time of *π*. Given periodicity *D* and place *p*, STP patterns are extracted from $q_{D,p,t}$ by finding the time segments whose associated probabilities are greater than a certain threshold, *θ*. Detailed descriptions on how to extract the STP patterns are shown in Algorithm 2. 

**Algorithm 2:** Algorithm for STP Pattern Extraction.
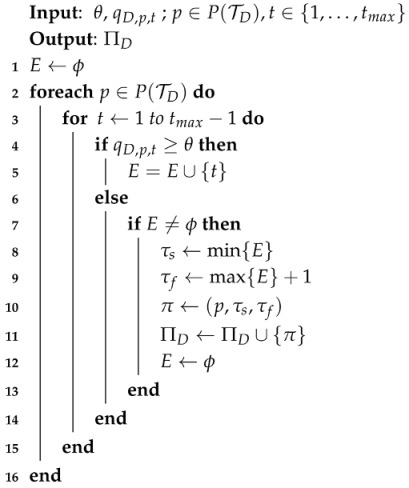


In Lines 3 to 15 of Algorithm 2, STP patterns are identified only for the consecutive time epochs whose probabilities are greater than or equal to *θ*. *E* is a temporary variable that records the set of consecutive time epochs, such that $q_{D,p,t}\geq \theta$. When $q_{D,p,t}$ falls below *θ* and *E* is not empty, a new STP pattern is identified by respectively setting $\tau_{s}$ and $\tau_{f}$ to be the start and finish time of the new pattern in Lines 8 to 9. Then, the new pattern is added to $\Pi_{D}$. The above procedure is repeated for all $p\in P(T_{D})$, and the algorithm finally returns the set of extracted STP patterns, $\Pi_{D}$.

Table 3a shows an example of STP patterns extracted from the ST trajectories in Table 2 when $T_{\left\{Mo\right\}}=\left\{T_{1},T_{8},T_{15}\right\}$ and θ=0.5. In Table 3a, $\pi_{1}$ is extracted from $T_{1,1}$, $T_{1,3}$ and $T_{15,1}$, while $\pi_{3}$ is from $T_{1,5}$ and $T_{15,3}$. STP patterns, $\pi_{1}$ and $\pi_{3}$, are illustrated as shaded areas in Figure 2c. For instance, $\pi_{1}$ is extracted from $q_{\left\{Mo\right\},p_{2},t}$ as follows: the goal is to find the consecutive time segments that satisfy $q_{\left\{Mo\right\},p_{2},t}\geq \theta$. Since $q_{\left\{Mo\right\},p_{2},t}=\frac{2\times (1-0.05(900-t))}{3}$ and $q_{\left\{Mo\right\},p_{2},t}=\frac{2-0.05(t-1035)}{3}$ when t∈[880,900] and t∈[1035,1055], respectively, t∈[895,1045] satisfies $q_{\left\{Mo\right\},p_{2},t}\geq \theta$. Therefore, $\tau_{s}$ and $\tau_{f}$ of $\pi_{1}$ are set to 895 and 1045, respectively, and as a result, $\pi_{1}=\left(p_{2},895,1045\right)$.

**Table 3:** Examples of (**a**) STP patterns; and (**b**) STP trajectories.

(a)	
π	$\left(\tau_{s},\tau_{f},p\right)$
$\pi_{1}$	$\left(895,1045,p_{2}\right)$
$\pi_{2}$	$\left(1015,1175,p_{3}\right)$
$\pi_{3}$	$\left(1155,1245,p_{2}\right)$
...	
(**b**)	
s	**Sequence**
$s_{1}$	$\langle e_{s},\pi_{1},\pi_{2},\pi_{3},e_{f}\rangle$
$s_{2}$	$\langle e_{s},\pi_{2},e\left(p_{1}\right),e_{f}\rangle$
$s_{3}$	$\langle e_{s},\pi_{1},\pi_{3},e\left(p_{4}\right),e_{f}\rangle$
...	

Note that the existence of $T_{1,2}$ between $T_{1,1}$ and $T_{1,3}$ was ignored during the construction of $\pi_{1}$. This is due to the smoothing applied to the probability of a stay, $q_{\left\{Mo\right\},p_{2},t}$, after the finish time of $T_{1,1}$ and before the start time of $T_{1,3}$, resulting in the effect of treating the user’s stay at $p_{1}$ during 10 min as a temporary visit that is often observed while a user is moving to another location. Indeed, the smoothing allows us to effectively combine the multiple re-visits to the same place even though the time intervals of their stays do not overlap, while providing a means to deal with temporary or irregular visits.

### 2.4. Generation of STP Trajectories

The user’s movement pattern is represented as an STP trajectory that is generated from ST trajectories by utilizing the extracted STP patterns for the user. We let *s* denote an STP trajectory. *s* is a sequence consisting of symbols, each of which corresponds to an STP pattern or event. It starts with event $e_{s}$ and ends with event $e_{f}$, respectively indicating the start and finish of *s*. The set of *s*’s generated for weekly periodicity *D* is denoted as $S_{D}$.

*s* is constructed by replacing each stay in an ST trajectory with the STP pattern that is most similar to the ST trajectory while sequentially exploring each ST trajectory in the ascending order of time. The similarity between a stay and an STP pattern is calculated based on overlap between the time segments of the stay and the pattern.

Specifically, the similarity between $T_{i,*}$ and $\pi^{*}$, denoted as *t*-$sim(T_{i,*},\pi^{*})$, is defined as Equation (1).

(1) $$t-sim(T_{i,*},\pi^{*})=\frac{\text{length of overlap between time intervals of}\;T_{i,*}\;\text{and}\;\pi^{*}}{\text{length of time interval of}\;T_{i,*}}$$

Algorithm 3 shows the detailed procedure for generating a set of STP trajectories from $T_{D}$ and $\Pi_{D}$, given a threshold for pattern similarity, $\theta^{\prime }$. In Algorithm 3, 〈·〉 is used for representing a sequence, and ⊕ denotes an operator for the concatenation of two sequences.

**Algorithm 3:** Algorithm for STP Trajectory Construction.
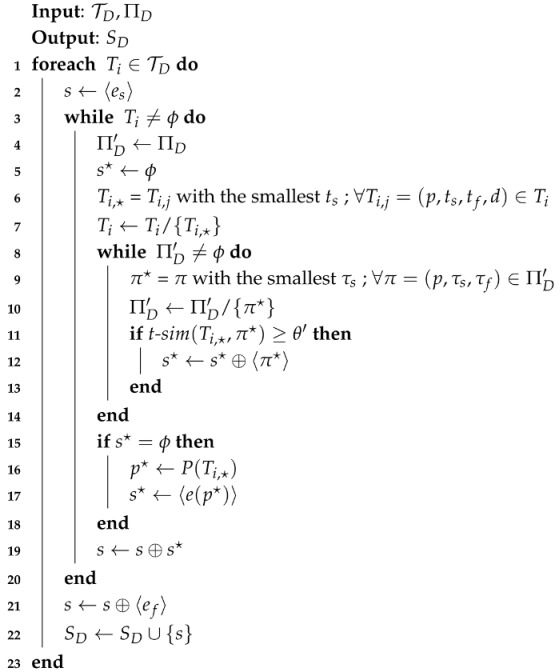


In Line 2, $e_{s}$ is added to *s* to represent the start of *s*, and in Line 6, stay $T_{i,*}$ with the smallest $t_{s}$ is selected to traverse in the ascending order of time and is removed from $T_{i}$ in the next line. From Line 8 to 14, the algorithm attempts to find the matching patterns for $T_{i,*}$ based on *t*-sim() among the candidate STP patterns by traversing the patterns in $\Pi_{D}^{\prime }$ one by one in the chronological order of $\tau_{s}$.

When there is no matching pattern found for $T_{i,*}$, the event of visiting place $P(T_{i,*})$, denoted as $e(p^{*})$, instead of an STP pattern is added to *s* as in Lines 15 to 18, where P() is a function that returns the place contained in stay $T_{i,*}$. The STP trajectory for $T_{i}$ is augmented with $s^{*}$ in Line 19, and $e_{f}$ is appended to *s* to indicate the end of the sequence in Line 21. Finally, the constructed STP trajectory *s* is added to $S_{D}$ as a member, and the algorithm returns $S_{D}$ as an output.

Table 3b shows an example of STP trajectory construction result from the ST trajectories in Table 2 by applying Algorithm 3 with the STP patterns defined in Table 3a. In Table 3b, STP trajectory $s_{1}$ consists of three STP patterns, $\pi_{1}$, $\pi_{2}$ and $\pi_{3}$. For constructing $s_{1}$, $\pi_{1}$ is selected as a matching pattern for $T_{1,1}$, since $\pi_{1}$ has the highest similarity among the STP patterns considered. Actually, *t*-$sim(T_{1,1},\pi_{1})$ was one as the time interval of $T_{1,1}$ is included in that of $\pi_{1}$. For $s_{3}$, an event of visiting place $p_{4}$ rather than an STP pattern is inserted at the fourth position, since there exists no matching pattern related to visiting $p_{4}$ around that time.

### 2.5. Gapped Sequence Mining

Among many sequential pattern mining algorithms that have been proposed in the past to discover frequent patterns from sequences, the gapped sequence mining algorithm has been known to provide satisfactory results in many applications [30]. It extracts patterns with consideration of gap constraints when finding frequent subsequences to relax the consecutiveness requirement on the subsequences. We employ a gap-constrained sequential pattern mining algorithm, known as cSPADE (Sequential Pattern Discovery using Equivalence classes with constraints) [27], to discover frequent subsequences from STP trajectories. It allows us to deal with irregular visits, as well as uncertainties in a mobile device log due to the presence of noisy data by using gap symbols.

Table 4a presents the result of applying the cSPADE algorithm to the STP trajectories in Table 3b. The outputs of the cSPADE algorithm are frequent subsequences with gaps, as well as their confidence values, which are then used to generate GSTP trajectories. The confidence of a sequence indicates the likelihood of occurrence of the last symbol in the sequence, given that all of the preceding symbols before the last one have been observed.

**Table 4:** Examples of the: (**a**) results of the cSPADE algorithm, as well as their confidences; and (**b**) GSTP trajectories.

(a)		
**No.**	**Subsequence with Gap**	**Confidence**
1	$\langle e_{s},\;\pi_{1}\rangle$	0.84
2	$\langle \pi_{3},\;e_{f}\rangle$	0.67
3	$\langle \pi_{1},\;\pi_{3},\;e_{f}\rangle$	0.75
4	$\langle \pi_{1},\;\pi_{3},\;e\left(p_{4}\right)\rangle$	0.78
5	$\langle \pi_{1},\;S_{g},\;\pi_{3},\;S_{g},\;e_{f}\rangle$	0.49
6	$\langle e_{s},\;\pi_{1},\;\pi_{2},\;\pi_{3},\;e_{f}\rangle$	0.55
7	$\langle e_{s},\;\pi_{1},\;\pi_{3},\;e\left(p_{4}\right),\;e_{f}\rangle$	0.27
...		
(**b**)		
σ	$\left(s^{p},s^{c},s^{s},u\right)$	
$\sigma_{1}$	$\left(\phi ,\;e_{s},\;\pi_{1},\;1.44\right)$	
$\sigma_{2}$	$\left(\phi ,\;\pi_{3},\;e_{f},\;1.27\right)$	
$\sigma_{3}$	$\left(\langle \pi_{1}\rangle ,\;\pi_{3},\;e_{f},\;1.25\right)$	
$\sigma_{4}$	$\left(\langle \pi_{1}\rangle ,\;\pi_{3},\;e\left(p_{4}\right),\;1.28\right)$	
$\sigma_{5}$	$\left(\langle \pi_{1},\;S_{g}\rangle ,\;\langle \pi_{3},S_{g}\rangle ,\;e_{f},\;0.89\right)$	
$\sigma_{6}$	$\left(\langle e_{s},\;\pi_{1},\;\pi_{2}\rangle ,\;\pi_{3},\;e_{f},\;1.15\right)$	
$\sigma_{7}$	$\left(\langle e_{s},\;\pi_{1},\;\pi_{3}\rangle ,\;e\left(p_{4}\right),\;e_{f},\;0.87\right)$	
...		

For instance, the fifth sequence in Table 4a is obtained from $s_{1}$ and $s_{3}$ of Table 3b, where $S_{g}$ denotes a set of sequences, each of which is an empty set or a sequence composed of events or patterns for representing a sequence of gap symbols. That is, subsequence of $s_{1}$, $\langle \pi_{1},\;\pi_{2},\;\pi_{3},\;e_{f}\rangle$, can be obtained from $\langle \pi_{1},\;S_{g},\;\pi_{3},\;S_{g},\;e_{f}\rangle$ by substituting the first $S_{g}$ with $\pi_{2}$ and the second $S_{g}$ with *ϕ*. Similarly, the subsequence of $s_{3}$, $\langle \pi_{1},\;\pi_{3},\;e_{f}\rangle$, can be generated by replacing all of the $S_{g}$’s with *ϕ*.

More formally, a GSTP trajectory, defined as four-tuple $\sigma =(s^{p},s^{c},s^{s},u)$, is obtained from a frequent subsequence in such a way that $s^{s}$ and $s^{c}$, respectively, are the symbols at the last and the second to last positions of the frequent subsequence, and $s^{p}$ corresponds to the rest. That is, a frequent subsequence found by cSPADE is split into three parts that respectively represent the current STP pattern or event (*i.e.*, $s^{c}$), the preceding patterns or events (*i.e.*, $s^{p}$) before $s^{c}$ and the succeeding pattern or event (*i.e.*, $s^{s}$) after $s^{c}$.

When $S_{g}$ is located at the second to last position in a frequent subsequence, its immediate predecessor together with $S_{g}$ form $s^{c}$, and all of the other predecessors constitute $s^{p}$. cSPADE is applied for each weekly periodicity *D* in D, and the resulting GSTP trajectories are stored into $\Sigma_{D}$.

Once GSTP trajectories are obtained, the average length of $S_{g}$ contained in $s^{p}$ of *σ* can be computed by counting the number of symbols corresponding to $S_{g}$ for each STP trajectory used to discover *σ* during the training process and taking their average. For instance, since $\sigma_{5}$ of Table 4b has been derived from $s_{1}$ and $s_{3}$ of Table 3b and $S_{g}=\left\{\pi_{2}\right\}$ for $s_{1}$ and $S_{g}=\phi$ for $s_{3}$, the average length of $S_{g}$ for $s^{p}$ of $\sigma_{5}$ is (1+0)/2=0.5. The average length of $S_{g}$ in $s^{c}$ can be computed in the same way.

Finally, u(σ) represents the utility of GSTP trajectory, *σ*, when making a prediction of the next place, and it is defined as Equation (2).

(2) $$\begin{array}{cc} u(\sigma )= & \;\left(\text{confidence of}\;\langle s^{p},s^{c},s^{s}\rangle \right)+\lambda {\left(1+\;\text{average length of}\;S_{g}\;\text{in}\;s^{p}\right)}^{-1}\\ & +\lambda^{\prime }{\left(1+\;\text{average length of}\;S_{g}\;\text{in}\;s^{c}\right)}^{-1} \end{array}$$

where *λ* and $\lambda^{\prime }$ are weight parameters.

u(σ) considers not only the confidence of a frequent subsequence, but also the average length of gaps located in $s^{p}$ and $s^{c}$ to accommodate the uncertainty associated with a GSTP trajectory. Note that the utility of a GSTP trajectory decreases as the gap symbols become longer. Furthermore, we set $\lambda^{\prime }$ to be greater than *λ* to put more emphasis on the utility related to the current and next places.

Table 4b presents an example of GSTP trajectories generated from the frequent subsequences in Table 4a, where *λ* and $\lambda^{\prime }$ were set to 0.1 and 0.5, respectively. There is a one-to-one correspondence between the subsequence of Table 4a and the GSTP trajectory of Table 4b. As an example, we consider the fifth subsequence in Table 4a, which is $\langle \pi_{1},\;S_{g},\;\pi_{3},\;S_{g},\;e_{f}\rangle$. $s^{s}$, $s^{c}$ and $s^{p}$ of $\sigma_{5}$ are $e_{f}$, $\langle \pi_{3},S_{g}\rangle$ and $\langle \pi_{1},S_{g}\rangle$, respectively, as $S_{g}$ is at the second to last position in the subsequence. Therefore, $u(\sigma_{5})$ is $0.49+0.1\times \frac{1}{1+0.5}+0.5\times \frac{1}{1+0.5}=0.89$.

### 2.6. Next Place Prediction

**Figure 3:**
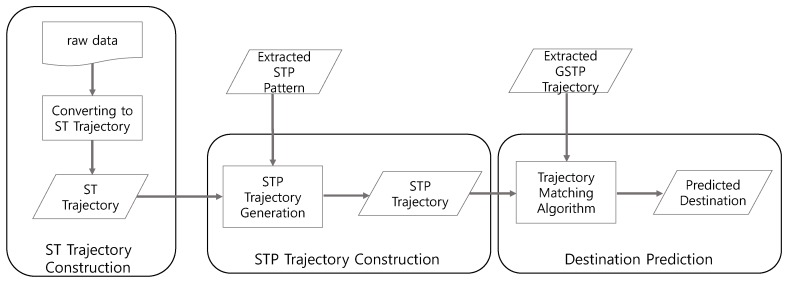
The test process for predicting next places based on raw test data.

Figure 3 depicts the test process for predicting the next location of a user when a new observation on the user’s movement is made. In order to predict the next place, it is necessary to convert the user’s movement logs to an STP trajectory and then to compare it to the GSTP trajectories identified during the training process. The steps involved in the test process are exactly the same as those in the training process in Figure 1, except for skipping the STP pattern extraction step for generating an STP trajectory. Once an STP trajectory is obtained from the test data, the user’s next place is predicted by Algorithm 4, which finds the most similar GSTP trajectory to the STP trajectory and predicts the next location by following the GSTP trajectory found.

**Algorithm 4:** Algorithm for Next Location Prediction. **Input**: $\Sigma_{D},s_{A}$ **Output**: $p^{*}$1$s_{A}^{c}$ = symbol at the last position of $s_{A}$.2$s_{A}^{p}$ = sequence preceding $s_{A}^{c}$ in $s_{A}$.3Find $\sigma =(s^{p},s^{c},s^{s},u)$ with the highest *p*-$sim(s^{p},s_{A}^{p})$ s.t. $s^{c}∋s_{A}^{c}$ ; $\forall \sigma \in \Sigma_{D}$4The ties are broken by picking *σ* with the highest *u*.5$p^{*}\leftarrow \;\text{place}\;p\;\text{of}\;s^{s}$

Algorithm 4 describes how the proposed framework infers the next place from an STP trajectory of user *A*, based on input data $s_{A}$, which is the STP trajectory of user *A* given as test data and $\Sigma_{D}$, GSTP trajectories constructed during the training process. $s_{A}$ is split into $s_{A}^{c}$ and $s_{A}^{p}$, which respectively are the last symbol that can be either an STP pattern or an event corresponding to the currently visiting place, and all of the symbols preceding $s_{A}$, denoting the past movements.

In Line 3 of Algorithm 4, the best matching STP pattern *σ* is found by examining the entire GSTP trajectories $\Sigma_{D}$. *σ*=$\left(s^{p},s^{c},s^{s},u\right)$ is obtained by use of the similarity between two sequences, *p*-$sim(s^{p},s_{A}^{p})$, that measures the length of overlapping subsequences between the sequences corresponding to the past movements. The similarity function is defined in Equation (3) in which $S_{g}$ is counted as of a length of one when calculating the length.

(3) $$p-sim(s^{p},s_{A}^{p})=\frac{\text{length of the intersection between}\;s^{p}\;\text{and}\;s_{A}^{p}}{\text{length of}\;s_{A}^{p}}$$

When there exist more than one STP pattern with same similarity value, the tie is broken by picking *σ* with the highest *u*. The algorithm then returns $p^{*}$, which is the place contained in $s^{s}$ representing a pattern or an event. Finally, execution of Algorithm 4 is repeated for all D∈D to select the best *σ* across the various weekly periodicities.

As an example, we assume $s_{A}$ = $\langle \pi_{1},\pi_{3}\rangle$, implying that user *A* is currently at $p_{2}$ (from Table 3a). Among the GSTP trajectories in Table 4b, $\sigma_{3}$ and $\sigma_{4}$ have the highest similarity, 1.0, as $s_{A}^{p}=\langle \pi_{1}\rangle$ is the same as $s^{p}$’s of $\sigma_{3}$ and $\sigma_{4}$. Between $\sigma_{3}$ and $\sigma_{4}$, we choose $\sigma_{4}$, since $u\left(\sigma_{4}\right)>u\left(\sigma_{3}\right)$. Accordingly, $p_{4}$ is predicted to be the user *A*’s next place, as $e(p_{4})$ of $s^{s}$ of $\sigma_{4}$ indicates an event of visiting place $p_{4}$.

## 3. Experiments

### 3.1. Dataset

Among several types of mobile devices, we adopted smartphones as data collection devices, since they are equipped with WiFi sensors and frequently carried by users anywhere they go throughout their daily activities. For experimentation, we implemented an Android mobile app that records the data pertaining to user’s visits, such as timestamps and WiFi signals, every minute. The mobile app was then distributed to eight students at Seoul National University (SNU), and the data were collected during two months spanning from September to November 2013.

The subjects were chosen in such a way that they have different majors; half of them are residents of a campus dormitory; and half of them take classes for more than 4 days a week, so that they can represent different campus lifestyles. As all of the participants were undergraduate students and the experiments were conducted during a semester, most activities they performed during the study period were related to typical campus life, including having a meal at a cafeteria, taking a class in a classroom, sleeping in a dormitory, doing homework in the library and doing exercise at a gym.

Since our research was a part of a smart campus project that aims to study intelligent services facilitating better campus life, data collection was conducted only inside the SNU campus, and all of the places considered were located within the campus. Another reason for limiting the scope to the SNU campus only was due to the availability of the WiFi fingerprinting database required by the proposed approach. Building a WiFi fingerprinting database involves time-consuming tasks and is very costly, but only the database for the SNU campus was available at the time of this research.

Throughout the experiments, all participants were instructed to carry their mobile devices with them as much as possible to gather comprehensive data that can reflect their actual daily movements. The full dataset contains 714,448 WiFi signal logs, and 19.85 WiFi APs were detected on average for each observation. Since the logs also include locations outside campus, only about 52 percent of logs were successfully mapped into meaningful places based on a localization method using the WiFi fingerprinting database for campus buildings. Furthermore, the first 42 days’ logs out of 60 days were selected as training data for constructing the prediction model, and the rest was used for evaluating the model’s performance.

**Figure 4:**
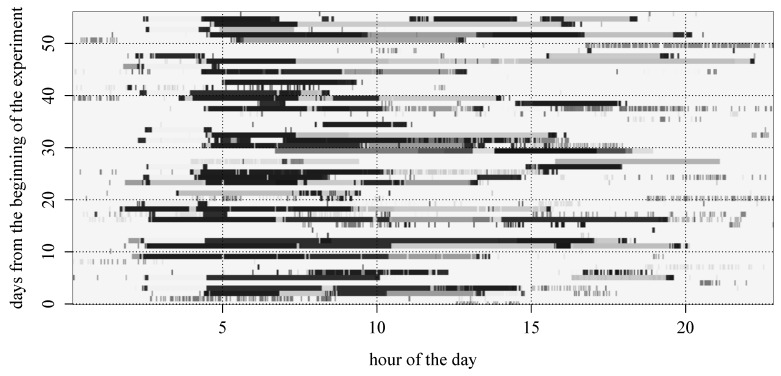
Visualization of a subject’s ST trajectories where blocks of the same gray level indicate the visits to the same place and white backgrounds represent unknown locations.

Figure 4 shows an example of a subject’s ST trajectories retrieved from the collected data. In this figure, blocks of the same gray level indicate visits to the same place, and white backgrounds represent unknown locations. The horizontal axis corresponds to the time from 0:00 to 24:00 of a day, while the vertical axis represents the number of days from the beginning of the experimentation. That is, the horizontal block stands for the subject’s stay at some place from the time at which the block begins until the time at which the block ends, and appearances of the blocks with the same gray level along with the vertical axis indicate that the subject visited the same place at similar time slots across the days.

From Figure 4, it can be observed that frequent revisits to the same place were usually made with weekly periodicities rather than daily due to the characteristics of campus life, and accordingly, we have extracted patterns based on the weekly periodicity. Yet, there are many irregular or exceptional visits that can be attributed to noisy observations, errors during localization or participant’s peculiarities, making the problem of next place prediction difficult. We address this difficulty by use of smoothing for constructing STP patterns and also by applying gapped sequence mining during the generation of GSTP trajectories.

### 3.2. Parameter Settings

**Figure 5:**
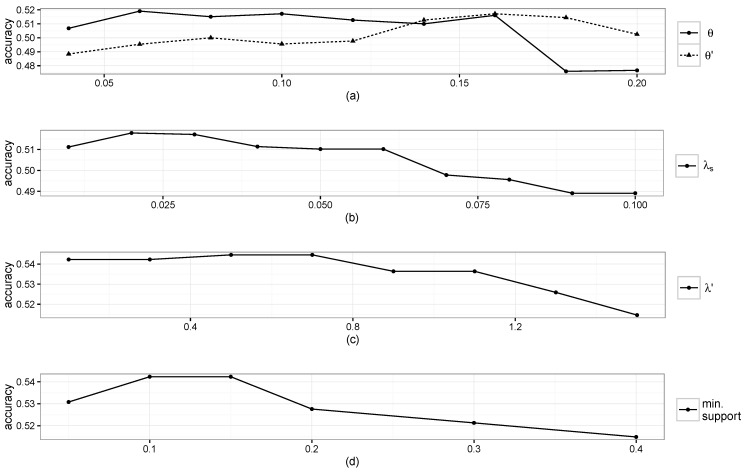
Accuracy results when varying parameters: (**a**) *θ* and $\theta^{\prime }$; (**b**) $\lambda_{s}$; (**c**) $\lambda^{\prime }$; and (**d**) minimum support.

For STP pattern extraction and STP trajectory construction, the parameters were determined experimentally by taking the values that maximize the performance of the proposed model. Figure 5a,b shows the prediction accuracy results when varying *θ*, $\theta^{\prime }$ and $\lambda_{s}$ individually while the other parameters were fixed. From Figure 5a,b, it can be seen that large *θ* hurts the performance as more false STP patterns are introduced, and roughly 50 min of smoothing are appropriate for identifying a stay. The highest performance was achieved when we respectively set *θ*, $\theta^{\prime }$ and $\lambda_{s}$ to 0.06, 0.16 and 0.02.

The maximum gap, maximum window size, minimum support, *λ* and $\lambda^{\prime }$ are the parameters involved in the gapped sequence mining. Individual effects of $\lambda^{\prime }$ and the minimum support on the accuracy are respectively plotted in Figure 5c,d, where the maximum performance was achieved when setting $\lambda^{\prime }$ and the minimum support to 0.5 and 0.15, respectively. Performance differences were negligible when varying the values of the maximum gap, maximum window size and *λ*, and we set them to 3, 7 and 0.1, respectively.

Finally, several weekly periodicities were selected in consideration of the characteristics of campus life, which include the periodicities based on a single day, except Saturday and Sunday, and those based on typical class schedules at SNU, resulting in D = {{Mo}, {Tu}, {We}, {Th}, {Fr}, {Mo,We}, {Tu,Th}, {Mo,We,Fr}}.

### 3.3. Evaluation Results

In order to demonstrate the effectiveness of the proposed framework, we have implemented two first-order Markov chain-based methods that predict the next place by calculating the probabilities for all of the possible next places based on the transition probabilities among places and choosing the place with the highest probability. We remark that the same ST trajectory data (like those in Table 2) were used for both the proposed methods and the first-order Markov chain methods to be fair with the presence of noisy data in the comparison.

**Figure 6:**
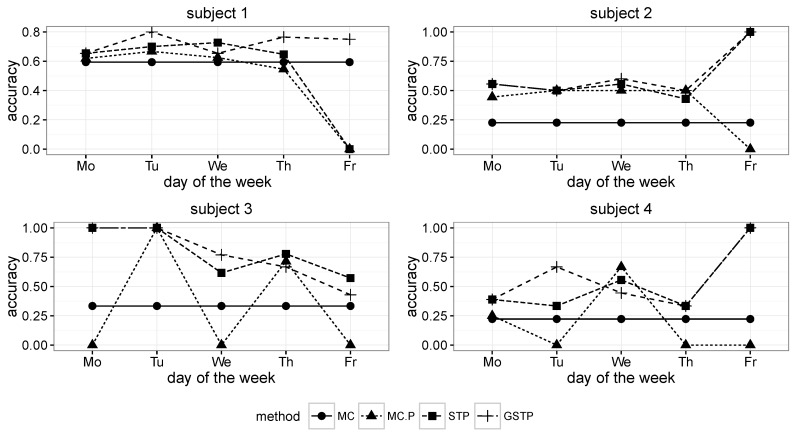
Accuracy results across the days of the week for Subjects 1 to 4. MC, Markov chain; P, periodicity.

**Figure 7:**
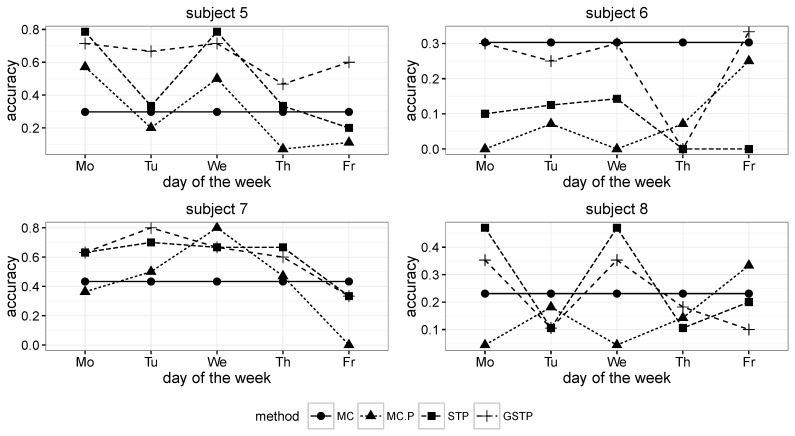
Accuracy results across the days of the week for Subjects 5 to 8.

The comparison results for the proposed methods and the Markov chain methods in terms of the accuracy metric are presented in Figure 6 and Figure 7, in which MC, MC-P, STP and GSTP, respectively stand for (1) the Markov chain method without periodicity consideration; (2) the Markov chain method with periodicity consideration; (3) the prediction based on STP trajectories; and (4) the prediction based on GSTP trajectories.

While MC predicted the next locations by using all of the available ST trajectory data without taking the day of week information into account, MC-P exploited the day of week information by selectively utilizing ST trajectories grouped by weekly periodicities according to the day on which prediction was made. Since MC prediction was performed on all of the trajectories in the training data, its accuracy results are the same across the day of the week, as shown in Figure 6 and Figure 7.

On the other hand, STP is based on the STP trajectory data (e.g., Table 3b) for the prediction that was made by choosing the pattern or event that has the highest transition probability from a current pattern or event after computing the transition probabilities between patterns or events. Finally, GSTP trajectories (e.g., Table 4b) were used for predicting the next location with the GSTP method.

**Figure 8:**
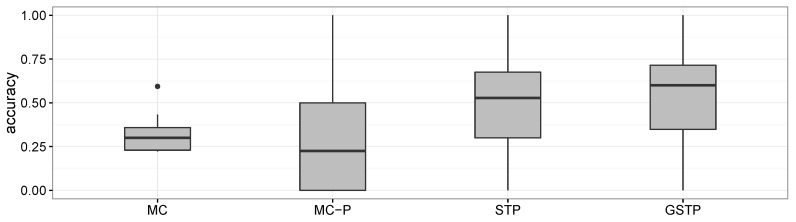
Boxplots for the accuracy results of the methods compared.

As shown in Figure 8, the overall accuracy results of MC and MC-P were worse than those of STP and GSTP. These poor performances yielded by the Markov chain-based methods are due to their inability to address the irregularities of visits, which is the characteristic often observed in campus life. In particular, the performance results of MC-P imply that the periodicity alone cannot help with increasing the accuracy. Figure 8 also shows that GSTP slightly outperformed STP on average, while their performance variabilities barely differ. Furthermore, it can be observed from Figure 8 together with Figure 6 and Figure 7 that STP and GSTP tend to provide more stable performances across the different days of the week than MC-P.

The next places were far from being predictable for some subjects, owing to the high irregularity in visiting behaviors when the MC and MC-P were used, but the prediction performances were greatly improved for them when applying the proposed methods, STP and GSTP. In particular, STP and GSTP significantly outperformed MC and MC-P for Subjects 3–5, as shown in Figure 6 and Figure 7.

Accordingly, it appears that the proposed notion of STP trajectory facilitates accuracy enhancement through generalizing observations into patterns, as well as accommodating periodicities. In addition, incorporation of gaps into the pattern sequence by the GSTP method was also successful for further increasing the accuracy. These together imply that the proposed framework was effective at predicting the user’s next location.

### 3.4. Effects of Movement Regularity

Besides the overall accuracy, we found out that the performance of the proposed methods significantly varied depending on the lifestyle of a subject. After the data collection experiment, we conducted a short survey asking about the regularity assessment for the subject’s movements during the study period in terms of a 3-point Likert scale. A score of 3 was reported by Subjects 1–3, indicating that they managed highly regular life patterns. On the other hand, the score of Subject 5 was 1, whereas the score of the rest was 2.

Based on this survey result, it appears that the performance of the proposed method was satisfactory when a subject exhibited highly regular behaviors, leading to the average prediction accuracy of more than 0.7 for Subjects 1 and 3. In contrast, when the visiting behavior of a subject was not very regular, the prediction performances of STP and GSTP were low, as suggested by the results for Subjects 6 and 8.

**Figure 9:**
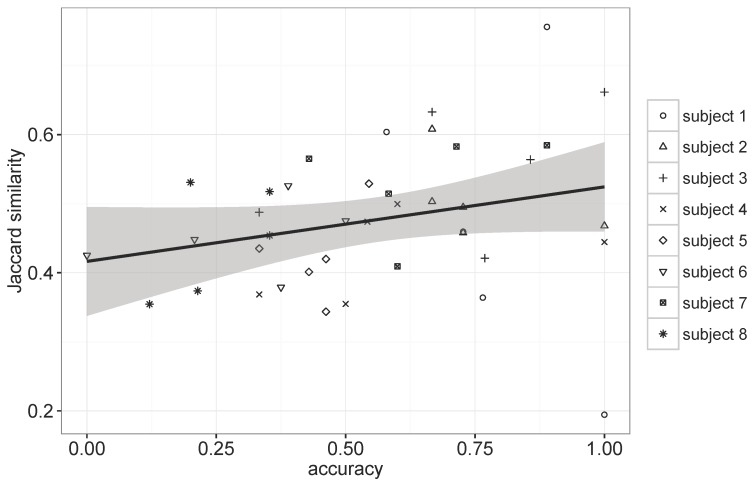
Scatter plot between the Jaccard similarity and the accuracy of GSTP.

To further explore the relationship between the regularity of movements and the prediction performance, we computed Jaccard similarity [31], which measures overlaps among the visited places by a subject for each day of the week, and employed it as a metric for assessing the regularity. Figure 9 shows the result that contains 40 plots corresponding to five different days of the week for eight subjects and their resulting performances.

The regularity score varied according to the subject, as well as the day of the week. The Pearson correlation coefficient for the plots in Figure 9 was 0.267, indicating a weak positive relationship between the regularity and the prediction accuracy of GSTP, which suggests that the regularity alone cannot fully explain the prediction performance due to the GSTP’s ability of accommodating irregularities through the smoothing and gapped sequence mining. It is still interesting to note that we can observe more dots in the upper right corner of Figure 9 for Subjects 1 to 3, who reported the highest scores for their subjective regularity assessment than for the other subjects, and *vice versa*.

## 4. Conclusions

In this paper, we exploited time, location and periodicity information to effectively predict the user’s next place through introducing the notion of the STP pattern and the application of gapped sequence mining. Frequently- and periodically-observed visiting behaviors were recognized as STP patterns for a user, and the patterns were then used for representing the user’s past visits as STP trajectories. Subsequently, the extracted STP trajectories were further generalized to GSTP trajectories to accommodate irregularities of visits, as well as to deal with exceptional stays.

Through the experimentation based on a real-world dataset collected from eight people, it was found that the proposed methods outperform the conventional methods based on the Markov chain in terms of prediction accuracy.

As future work, we plan to apply our work to larger and more complex environments than a university campus, such as urban areas or travel sites with more participants, and to further enhance the proposed spatiotemporal-periodic patterns through developing more sophisticated similarity measures that can effectively accommodate diverse types of irregularities and the semantic meaning of places.
